# Psychological distress 35 years after the Chornobyl accident in the Lithuanian clean-up workers

**DOI:** 10.1080/16549716.2023.2233843

**Published:** 2023-07-17

**Authors:** Evaldas Kazlauskas, Giedre Smailyte, Ingrida Domarkienė, Vaidutis Kučinskas, Aušra Matulevičienė, Ask Elklit, Gabrielė Žukauskaitė, Laima Ambrozaitytė

**Affiliations:** a Center for Psychotraumatology, Institute of Psychology, Vilnius University, Vilnius, Lithuania; b Laboratory of Cancer Epidemiology, National Cancer Institute, Vilnius, Lithuania; c Institute of Health Sciences, Faculty of Medicine, Vilnius University, Vilnius, Lithuania; d Institute of Biomedical Sciences, Faculty of Medicine, Vilnius University, Vilnius, Lithuania; e Danish National Centre for Psychotraumatology, Department of Psychology, University of Southern Denmark, Odense, Denmark

**Keywords:** Chornobyl, disaster, clean-up workers, psychological distress, depression, anxiety, Lithuanian population

## Abstract

The adverse effects on the health of the Chornobyl nuclear power plant accident clean-up workers have been reported previously. However, there is a lack of studies on the mental health of Chornobyl clean-up workers. The current study explored psychological distress in a sample of Lithuanian clean-up workers 35 years after the accident. In total, 107 Lithuanian Chornobyl clean-up workers (M_age_ = 62.5) and 107 controls were included in the study. The Hospital Anxiety and Depression Scale (HAD) was used for the assessment of anxiety and depression. The depression symptoms were significantly higher in the clean-up workers compared to the control group. The prevalence of severe depression symptoms was 23.4% and 4.7% in the Chornobyl clean-up workers and control groups, respectively. The risk for severe depression was associated with Chornobyl clean-up work (adjusted OR = 5.9). No differences in the anxiety symptoms were found between clean-up workers and controls. The study revealed the deteriorated mental health of the Lithuanian Chornobyl clean-up workers 35 years after the disaster – in particular, high levels of depression. Psychosocial support programmes for clean-up workers should be provided to mitigate the adverse effects of the disaster.

## Introduction

Over half a million clean-up workers were recruited in disaster response in the contaminated area following the Chornobyl nuclear power plant accident on 26 April 1986 [1–3]. To this day, the Chornobyl accident is the largest industrial disaster in human history, and it had a profound effect on millions of the population in Ukraine and the region. Clean-up workers were mainly tasked to perform decontamination and construction work, and the majority were exposed to high dosages of ionising radiation, especially around 200 000 of those who worked in the years 1986–1987 in the 30 km zone surrounding the power plant. Activities in the accident zone had a prolonged effect on the Chornobyl clean-up workers or ‘liquidators’, a new term coined by Soviet officials following the incident. Studies on the health effects of the Chornobyl accident, reported long-term effects on the clean-up workers’ physical health, especially high cancer risk [1,4]. However, the aftermath of the Chornobyl disaster on mental health, although identified as a major public health concern [5], received little attention in research. Therefore, knowledge of the long-term psychosocial effects on clean-up workers of the Chornobyl accident is limited.

There are a few reports on the long-term negative effects of the Chornobyl accident on psychological distress and psychiatric disorders among clean-up workers [3]. One of the first long-term studies estimating the impact of the Chornobyl incident 8 years after the disaster, was reported in a sample of 1,412 Latvian clean-up workers, which found 44% of mixed mental-psychosomatic disorders [6]; however, no comparison with the general population sample was made in the study. A study in Ukraine reported elevated rates of depression (14.9% vs. 7.1%) and post-traumatic stress disorder (PTSD) (4.1% vs. 1.0%) among 295 clean-up workers compared to controls 18 years after the Chornobyl accident [7]. In another study, depression and anxiety symptoms were significantly higher among 614 Estonian Chornobyl clean-up workers than among controls at a 24-year follow-up after the accident [8]. Further investigation of 99 Chornobyl clean-up workers from Estonia in the same sample confirmed the increased risk of mental disorders – in particular, depression (OR = 3.07), alcohol dependence (OR = 3.47), and suicide ideation (OR = 3.44) [9].

Considering the scope and impact of the Chornobyl disaster, the mental health aftermath on the clean-up workers has been poorly studied, and more research is highly needed. The current study explored the mental health in a Lithuanian sample of Chornobyl accident clean-up workers – which had not been studied and reported previously. At the time of the accident, clean-up workers were brought into the disaster site from various regions of the Soviet Union. Back then, Lithuania was a part of the Soviet Union, and it is estimated that around 7,000 individuals were sent to Chornobyl from 1986 to 1990 [10]. Lithuanian clean-up workers were military reservists who were active in the power plant or the surrounding area and were exposed to high doses of radiation [11]. The aim of this study was to evaluate anxiety and depression in a sample of the Lithuanian Chornobyl accident clean-up workers.

## Methods

### Participants

A total of 107 Lithuanian males who were recruited to work at the Chornobyl nuclear power plant accident zone participated in this study. These participants had an officially recognised status of Chornobyl clean-up workers based on the legislation of Lithuania. The work mainly included clean-up and construction work in the disaster area in the first four years after the disaster. The control group comprised 107 Lithuanian males representing individuals from the general population. The groups were matched in education, rural/urban location, and age range (see Table 1). Data was collected from 2019 to 2022.

**Table 1.** Characteristics of the sample groups.

Variable	Chornobyl clean-up workers (*n* = 107)	Control group *(n* = 107)	Significance statistics
Gender			
Male	107 (100%)	107 (100%)	-
Age			
*M* (*SD*)	62.51 (6.05)	56.98 (5.69)	*t*(212) = 6.89, *p* < .001
Range	49–76	49–78	
Median	61	56	
Skewness	.45	1.38	
Kurtosis	−.38	2.33	
Education			
Primary	4 (3.3%)	3 (2.8%)	χ^2^(2) = 5.83, *p* = .120
Secondary	39 (36.8%)	24 (22.4%)	
Professional college	45 (42.5%)	59 (55.1%)	
University degree	18 (17.0%)	21 (19.6%)	
Place of residence			
Rural	68 (63.6%)	79 (73.8%)	χ^2^(1) = 2.63, *p* = .105
Urban	39 (36.4%)	28 (26.2%)	
HAD scores^a^			
Anxiety	6.17 (SE = .38)	6.30 (SE = .38)	*F*(1,211) = 0.05, *p* = .823
Depression	7.41 (SE = .39)	5.50 (SE = .39)	*F*(1,211) = 10.93, *p* = .001
Anxiety			
None (HAD-A ≤7)	65 (60.7%)	78 (72.9%)	χ^2^(2) = 3.56, *p* = .168
Moderate (HAD-A = 8–10)	23 (21.5%)	16 (15.0%)	
Severe (HAD-A ≥11)	19 (17.8%)	13 (12.1%)	
Depression			
None (HAD-D ≤ 7)	56 (52.3%)	79 (73.8%)	χ^2^(2) = 17.13, *p* < .001
Moderate (HAD-D = 8–10)	26 (24.3%)	23 (21.5%)	
Severe (HAD-D ≥ 11)	25 (23.4%)	5 (4.7%)	

HAD = Hospital Anxiety and Depression Scale, ^a^HAD estimated means, after controlling for age.

### Assessments

A self-report Hospital Anxiety and Depression Scale (HAD) was used to assess anxiety and depression symptoms [12]. The HAD scale comprises 14 items, with two subscales measuring anxiety (HAD-A, 7 items) and depression (HAD-D, 7 items). Participants were asked to respond to each item on a 4-point Likert scale ranging from 0 to 3. The scores for both anxiety and depression subscales range from 0 to 21. The HAD-A and HAD-D score ≤7 indicates no anxiety and depression, 8–10 moderate symptoms, and >10 severe anxiety or depression. The HAD scale has been widely used in various samples in Lithuania (e.g. [13]).

### Data analysis

A chi-square test was used to estimate group effects on the prevalence of anxiety and depression in the sample group. A univariate ANCOVA test was applied to compare anxiety and depression symptoms. Binary logistic regression analysis was used to evaluate the risk for high levels of anxiety and depression associated with exposure to Chornobyl clean-up work. Data analysis was conducted using SPSS ver. 28.

## Results

Depression symptoms were higher in the Chornobyl disaster clean-up workers group (See Table 1) than in the control group, controlling for age. Further, we found significant differences in depression levels between groups. Around half of the Chornobyl clean-up workers (47.7%) had moderate-to-severe depression vs. 26.2% in the control group. Furthermore, severe depression symptoms were 23.4% and 4.7% in the Chornobyl clean-up workers and the control group, respectively. However, no statistical differences were found in anxiety symptoms between the clean-up workers group and the controls. No significant age or education-level effects on anxiety and depression in the clean-up workers sample were found.

Further, we conducted a binary logistic regression analysis to estimate how work in the Chornobyl accident area predicted psychological distress after controlling for age and education. We found that exposure in the Chornobyl area significantly predicted severe depression symptoms (adjusted OR = 5.87, *p* = .001) but not anxiety (adjusted OR = 1.38, *p* = .590).

## Discussion

In this first study, which explored mental health in the Lithuanian Chornobyl accident clean-up workers, we found high levels of depression symptoms compared to the control group. Moreover, the prevalence of moderate-to-severe depression (47.7%), and only severe depression (23.5%) symptoms in our clean-up sample was higher than in other Lithuanian studies that utilised HAD to screen depression risk. Previous studies in Lithuanian samples reported 13.5% of severe depression symptoms (HAD-D > 10) in the general population aged 60–84 [14], and 22.8% moderate-to-severe depression symptoms (HAD-D > 7) in primary healthcare patients aged 18–87 [13], and 30% moderate-to-severe depression symptoms (HAD-D > 7) in a population-based sample aged 62 years on average [15]. Our study findings are in line with previous studies which reported the poor mental health of Chornobyl disaster clean-up workers in Ukraine [7] and other Baltic countries, namely Estonia [8] and Latvia [6]. However, our study adds to the existing body of evidence of the Chornobyl accident’s effects on mental health by providing data in a new sample group and longer follow-up after the disaster.

Poor mental health and high levels of depression symptoms in the sample of clean-up workers can be associated with the effects of ionising radiation on health, but also social factors. In particular, the low recognition of the impact of disasters on mental health in the region [16]. During the Soviet Era, when the Chornobyl disaster happened, psycho-social support was not included in emergency responses and disaster management [17]. Furthermore, there was much secrecy around the impact of the disaster on the general population and clean-up workers. Clean-up workers were sent to the nuclear disaster without warning, training, or consent. Being military reservists at the time, they often were not aware of why and where they were sent. After the collapse of the Soviet Union, Lithuania gained independence, and mental health services developed significantly over several decades. However, no systematic, sustainable, long-term mental healthcare services were provided to the Chornobyl clean-up workers. Moreover, to this day, no mental health intervention trials have been conducted on the Chornobyl clean-up workers or other populations affected by this disaster [17].

The study has several limitations; therefore, our results should be interpreted with caution. First, a sample group of Lithuanian clean-up workers was not randomly selected. The registry of all Lithuanian clean-up workers is not maintained or updated, and for this reason is not available to researchers. Second, we aimed to recruit a matched group from the general population. The control group data were extracted from the LITGEN project, which explored the genomes of Lithuania’s general population. While this is the comparable cohort of men to the clean-up workers included in the study, it was not a representative sample, and the mean age in the group was lower by several years compared to clean-up workers.

Furthermore, it is possible that higher levels of depression symptoms were associated with the long-term effects of radiation on health. Therefore, future studies should explore the links between medical illness and mental health in Chornobyl clean-up workers. Finally, we do not have baseline data, nor could we monitor mental health changes using the multiple time points across several decades in clean-up workers following the accident. As was mentioned previously, the specific social context banned initiatives to launch mental health research during the Soviet Era, and rapid social changes after the collapse of the Soviet Union hindered research in the area, and was not a priority when dealing with atrocities of the past.

## Conclusion

Despite the limitations, this study provides important data on the long-term adverse effects of the Chornobyl disaster on clean-up workers. The study found high levels of depression in the Lithuanian clean-up workers 35 years after the Chornobyl accident. The adjusted odds ratio indicated the risk of having severe depression levels in the clean-up workers group was 5.9 times higher in contrast to the control group. Future health studies of the Chornobyl disaster should address the impact of the disaster on mental health and include mental health measures. The study informs that specialised intervention programmes should be considered to address the clean-up workers’ specific needs to reduce the disaster burden on their mental health. Evaluating the outcomes of such intervention programmes, could provide valuable insights and a better understanding of how to mitigate the negative effects on the mental health of the Chornobyl clean-up workers and survivors of other disasters who did not receive mental health services for decades following a disaster.

## Paper context

The Chornobyl nuclear power plant accident on 26 April 1986 was one of the largest industrial disasters in human history. Over half a million workers were recruited in disaster response in contaminated areas following the Chornobyl disaster. The accident had a long-term effect on the health and mental health of the disaster clean-up workers. The current paper reveals the poor mental health of clean-up workers 35 years after the disaster.
